# Erfolgreiche Behandlung einer chronischen Prurigo mit Dupilumab

**DOI:** 10.1007/s00105-020-04721-0

**Published:** 2020-11-12

**Authors:** Julia K. Winkler, Holger A. Haenssle, Alexander Enk, Ferdinand Toberer, Martin Hartmann

**Affiliations:** Universitäts-Hautklinik Heidelberg, Ruprecht-Karls Universität Heidelberg, Im Neuenheimer Feld 440, 69120 Heidelberg, Deutschland

**Keywords:** Prurigo nodularis, Atopisches Ekzem, Atopische Diathese, Lebensqualität, Genese, Nodular prurigo, Atopic eczema, Atopic diathesis, Quality of life, Etiology

## Abstract

Chronische Prurigo ist durch anhaltenden Pruritus, teils einhergehend mit sekundären Kratzläsionen, gekennzeichnet. Die Abklärung der Genese ist von besonderem Stellenwert, wobei eine atopische Diathese häufig einen ätiologischen Faktor darstellt. Wir präsentieren einen Patienten mit chronischem Pruritus multifaktorieller Genese (atopische Diathese, chronische Niereninsuffizienz, Diabetes mellitus, Polyneuropathie). Nach multiplen erfolglosen Vortherapien behandelten wir den Patienten mit Dupilumab, worunter sich ein sehr positiver Erkrankungsverlauf mit deutlicher Besserung der Lebensqualität zeigte.

## Abstract (en)

Chronic prurigo is characterized by persistent itching und partly accompanied by secondary skin excoriation. Diagnostic evaluation is of special relevance and atopic diathesis is a frequent pathogenic factor. We present a patient with prurigo of multifactorial etiology (atopic diathesis, impaired kidney function, diabetes and polyneuropathy). After several unsuccessful prior treatment approaches, the patient was treated with dupilumab, which resulted in a tremendous improvement of itching, skin lesions, and quality of life.

## Anamnese

Ein 83-jähriger Patient stellte sich mit massivem Pruritus in unserer allgemeinen Ambulanz vor. Der Pruritus wurde als quälend und schmerzhaft beschrieben mit einer Intensität von 10/10 auf der visuellen Analogskala (VAS). Der Pruritus habe bereits seit mehreren Jahren bestanden, zuletzt jedoch an Intensität zugenommen. Der Patient war bei Vorstellung durch Schlaflosigkeit und Ruhelosigkeit aufgrund des Juckreizes massiv beeinträchtigt und im Alltag belastet und zeigte sich deutlich Hilfe suchend. Bekannte Nebenerkrankungen waren eine chronische Niereninsuffizienz im Stadium IV bei Nephrosklerose bzw. diabetischer Nephropathie, ein Diabetes mellitus Typ 2, eine arterielle Hypertonie, ein Tremor (Differenzialdiagnose: Morbus Parkinson) und eine Hyperurikämie. Sitagliptin und Candesartan wurden als Dauermedikation eingenommen. Eine Rhinitis allergica, ein allergisches Asthma oder ein atopisches Ekzem in der Kindheit wurden verneint.

## Klinischer Befund

**Figure 1:**
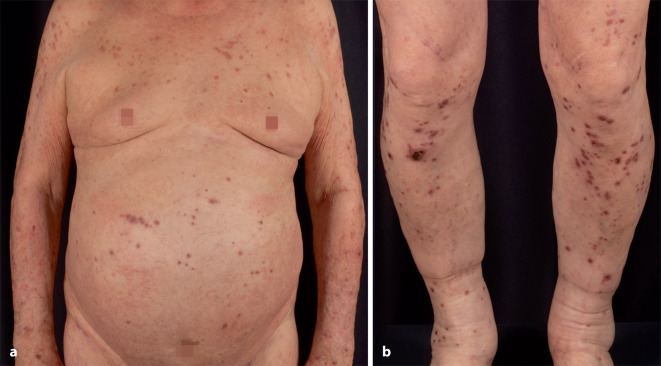

Die dermatologische Inspektion zeigte im Bereich des Stamms und der Extremitäten multiple erythematös livide Papeln mit zentraler Kruste oder Exkoriation (Abb. 1). Der mittlere obere Rücken war weitgehend von Hautveränderungen ausgespart. Insbesondere an den streckseitigen Unterschenkeln zeigten die Läsionen teils zentral kraterförmig eine dunkle, festhaftende Keratose (Abb. 1b). Einige Läsionen imponierten im Sinne von Prurigoknoten, zum Teil fanden sich klinische Ähnlichkeiten mit einer reaktiven perforierenden Kollagenose. Am Abdomen zeigten sich zusätzlich einzelne flächig imponierende Erytheme (Abb. 1a).

**Figure 2:**
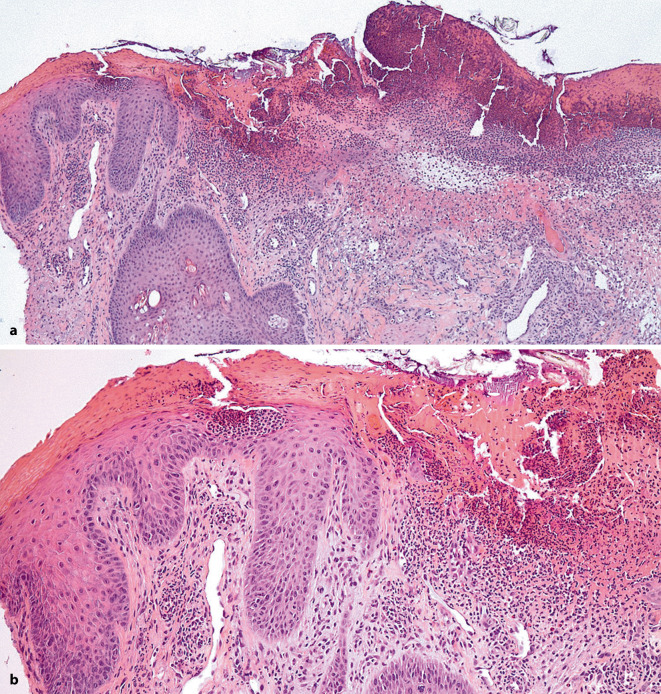

Es wurde eine Probebiopsie vom Unterarm entnommen. Die Histologie zeigte eine oberflächliche Ulzeration mit aufgelagerter Serumkruste und oberflächlich ein dichtes gemischtzelliges entzündliches Infiltrat (Abb. 2). Fokal zeigte sich auch gruppiert intraepidermal eine Ansammlung von Lymphozyten (Abb. 2b).

## Diagnose

Bei dem Patienten wurde bei chronischem Pruritus mit Dominanz sekundärer Kratzläsionen eine chronische Prurigo multifaktorieller Genese diagnostiziert. Bezüglich der Ätiologie der chronischen Prurigo wurden bei dem Patienten mehrere Faktoren diskutiert. Eine Niereninsuffizienz (GFR [glomeruläre Filtrationsrate] ca. 30 ml/min) und ein Diabetes mellitus waren vorbekannt, zudem wurde eine neuropathische Komponente im Sinne eines sekundär generalisierten Pruritus bei erosiver Osteochondrose HWK (Halswirbelkörper) 6/7 diskutiert. In der durchgeführten Laboruntersuchung zeigten sich zusätzlich Hinweise auf eine atopische Diathese bei deutlich erhöhtem Gesamt-Ig(Immunglobulin)E (>5000 kU/l, Referenz <100 kU/l) und positivem Inhalationsscreen (Sx1; 1,13 kU/l, Referenz <0,35 kU/l).

## Therapie und Verlauf

**Figure 3:**
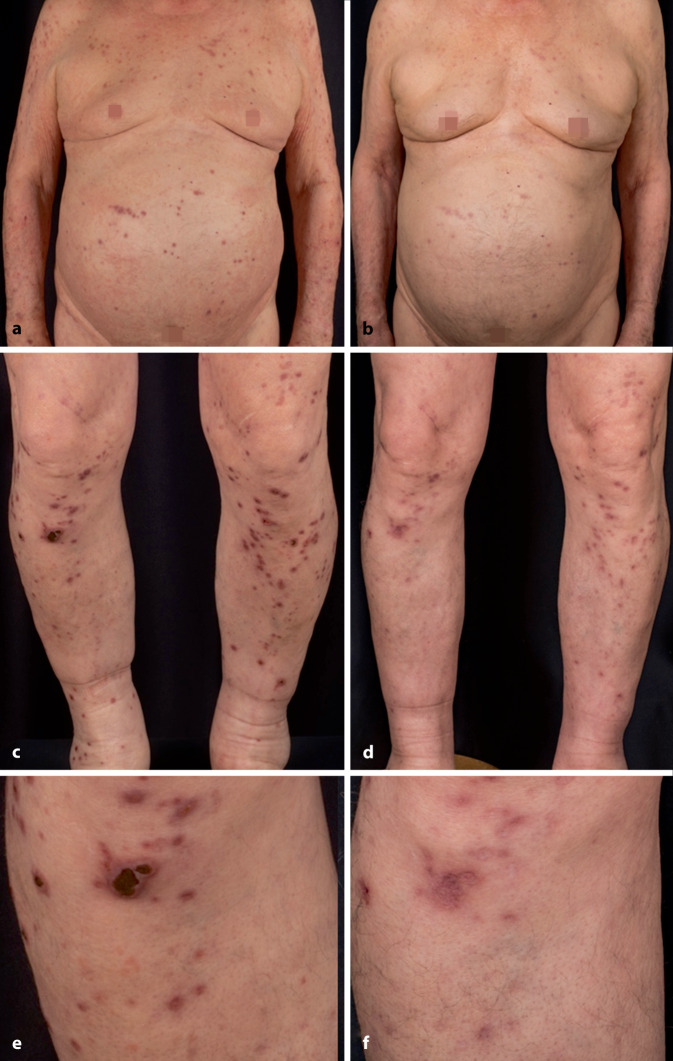

Bei Vorstellung hatte der Patient bereits multiple Vortherapien erhalten, unter anderem topische Steroide und Calcineurininhibitoren sowie Antihistaminika (Desloratadin und Dimetinden jeweils mehrfach täglich) sowie Gabapentin. Im Rahmen einer tagesklinischen Behandlung in unserer Klinik wurde die topische Therapie nochmals intensiviert. Es kamen topische Steroide der Klasse III und IV in pflegender Grundlage, zudem antipruriginöse Topika wie Polidocanol und probatorisch auch teerhaltige Externa zum Einsatz. Bei begrenzter Wirksamkeit wurde eine selektive Ultraviolett-Phototherapie(SUP)-Behandlung ergänzt. Die Therapie mit Gabapentin wurde auf Pregabalin umgestellt. Aufgrund des Pruritus und der Schlaflosigkeit zeigte der Patient eine Agitiertheit, sodass nach psychiatrischem Konsil eine Behandlung mit Pipamperon als sedierendes Neuroleptikum eingeleitet wurde. Im Rahmen eines individuellen Heilversuchs entschieden wir uns für eine Behandlung mit dem Neurokinin-1-Rezeptor-Antagonisten Aprepitant. Bei weiter quälendem Pruritus wurden zusätzliche Therapieoptionen evaluiert. Systemische Steroide, Ciclosporin und Methotrexat waren bei unserem Patienten aufgrund der bestehenden Begleiterkrankungen Diabetes mellitus und Niereninsuffizienz relativ kontraindiziert. Somit haben wir uns aufgrund des ausgeprägten Leidensdrucks für die Einleitung von Dupilumab entschieden. Es stellte sich rasch eine subjektive Besserung des Juckreizes ein, der nun mit Werten von 1 bis 2 auf der VAS angegeben wird. Auch die Prurigoläsionen zeigten sich im Verlauf von nur wenigen Monaten deutlich abgeflacht und gebessert (Abb. 3). Besonders eindrücklich rückläufig zeigten sich die festhaftenden Keratosen an den Unterschenkeln (Abb. 3e, f). Der Patient gab eine deutliche Besserung von Schlaf und Lebensqualität an. Die Therapie mit Dupilumab wurde zwischenzeitlich über nahezu 1 Jahr fortgeführt und ohne relevante Nebenwirkungen toleriert.

## Diskussion

Wir berichten einen positiven Krankheitsverlauf unter Dupilumab bei chronischer Prurigo [8]. Unser Patient war aufgrund des quälenden Pruritus massiv in seiner Lebensqualität beeinträchtigt. Die S2k-Leitlinie zur Diagnostik und Therapie des chronischen Pruritus liefert einen sehr guten Überblick über mögliche Therapieoptionen in Abhängigkeit der jeweils zugrunde liegenden Ätiologie [9]. Bei unserem Patienten waren die Therapieoptionen aufgrund bestehender Begleiterkrankungen insgesamt limitiert. Im Rahmen eines individuellen Heilversuchs wurde Aprepitant eingesetzt. Aprepitant wird in der deutschen Leitlinie als Therapieoption bei Prurigo nodularis oder paraneoplastischem Pruritus angeführt [9]. Eine Phase-2-Studie hatte zuletzt die Wirksamkeit von Serlopitant bei Prurigo nodularis im Vergleich mit Placebo gezeigt [10]. In Studien wird daher aktuell Serlopitant bei Prurigo nodularis weiter evaluiert (NCT 03677401). Da unser Patient kein ausreichendes Ansprechen auf Aprepitant zeigte, entschieden wir uns für eine Therapie mit Dupilumab. Im Allgemeinen wird bei chronischer Prurigo eine atopische von einer nichtatopischen Variante unterschieden, wobei laut Literatur bei nahezu 50 % der Patienten mit Prurigo nodularis eine atopische Komponente festgestellt werden kann [5]. Bei unserem Patienten gehen wir von einer multifaktoriellen Genese der chronischen Prurigo bei zugrunde liegender atopischer Diathese aus. Anhand der bestehenden Hautveränderungen ergaben sich keine hinreichenden Hinweise auf atopische Stigmata oder Minimalformen. Lediglich am Abdomen imponierten dezent flächige Erytheme. Sekundäre Kratzläsionen standen im Hinblick auf das klinische Bild deutlich im Vordergrund. Richtungsweisend bezüglich einer zugrunde liegenden atopischen Diathese war letztendlich der deutlich erhöhte Gesamt-IgE-Wert im Serum. Die Wirksamkeit von Dupilumab bei der Prurigovariante des atopischen Ekzems wurde bereits berichtet [6]. Auch bei den als Prurigo nodularis imponierenden, generell schwer zu behandelnden Varianten des atopischen Ekzems scheint Dupilumab ersten Berichten zufolge eine gute Wirksamkeit zu zeigen [3]. Unser Fallbericht verdeutlich eindrücklich, dass eine atopische Diathese bei Vorliegen einer Prurigoerkrankung expliziert evaluiert werden sollte, da diese nicht immer auf den ersten Blick klinisch anamnestisch festzumachen ist. Im vergangenen Jahr sind mehrere Fallberichte und kleinere Fallserien publiziert worden, die weiterhin eine Wirksamkeit von Dupilumab bei Prurigo nodularis verschiedenster Genese zeigten [1]. Die größte Fallserie aus Frankreich berichtet über 16 Patienten mit Prurigo nodularis, die von einer Behandlung mit Dupilumab profitierten [2]. Eine weitere Fallstudie konnte eine Wirksamkeit von Dupilumab sowohl bei Prurigo nodularis als auch bei anderen Formen des chronischen Pruritus unter anderem bei urämischem Pruritus zeigen [11]. Bezüglich des zugrunde liegenden Pathomechanismus wurde bei Prurigo nodularis epidermal vermehrt aktiviertes pSTAT6 gefunden [4]. Der pSTAT6-Signalweg wird aktiviert durch die Th2-Zytokine IL(Interleukin)-4 und IL-13, sodass geschlussfolgert wurde, dass Th2-Zytokine eine Rolle in der Pathogenese der Prurigo nodularis spielen [4]. Weiterhin wurde von Oetjen et al. gezeigt, dass Th2-Zytokine sensorische Neurone im Hinterhorn des Rückenmarks aktivieren, die für die Vermittlung von Pruritus von Bedeutung sind [7]. Sie konnten zeigen, dass die Depletion des IL-4α-Rezeptors bzw. von JAK(Januskinase)1 chronischen Pruritus reduziert [7]. Die Wirksamkeit von Dupilumab bei Prurigo nodularis wird aktuell in laufenden klinischen Studien evaluiert (NCT04183335).

Insgesamt verdeutlicht die dargestellte Kasuistik die komplexe diagnostische Abklärung und Behandlung eines Patienten mit chronischem Pruritus und Dominanz sekundärer Kratzläsionen. Ätiologische Abklärung und individuelle Therapieentscheidung sind bei chronischem Pruritus von besonderer Relevanz. In unserem Fallbericht präsentieren wir einen Patienten mit chronischer Prurigo, der relevant von Dupilumab profitieren konnte. Dupilumab sollte bei Patienten mit chronischer Prurigo als Therapieoption in Erwägung gezogen werden. Es ist von Interesse, Dupilumab im Hinblick auf seine Wirksamkeit bei chronischer Prurigo weiter zu evaluieren.

## Fazit für die Praxis

- Chronischer Pruritus kann ein für den Patienten extrem belastendes Symptom darstellen. Die Genese ist multifaktoriell, wobei eine atopische Diathese in der Ätiologie häufig eine Rolle spielt.
- Dupilumab zeigt nicht nur Wirksamkeit bei atopischer Dermatitis, positive Effekte sind auch bei Prurigo nodularis multifaktorieller Genese berichtet.
- Dupilumab sollte daher bei chronischem Pruritus als Therapieoption für Patienten mit ausgeprägtem Leidensdruck erwogen werden und in Studien weiter evaluiert werden.
